# Relationship of Natriuretic Peptides with Left Atrial Structure and Function within 1 Month after Electrical Cardioversion in Patients with Persistent Atrial Fibrillation

**DOI:** 10.1155/2019/7636195

**Published:** 2019-03-17

**Authors:** Rasa Karaliute, Justina Jureviciute, Julija Jurgaityte, Giedre Stanaitiene, Vaida Mizariene, Tomas Kazakevicius, Daiva Urboniene, Ausra Kavoliuniene

**Affiliations:** ^1^Laboratory of Behavioral Medicine, Neuroscience Institute, Medical Academy, Lithuanian University of Health Sciences, Kaunas LT-50161, Lithuania; ^2^Department of Cardiology, Medical Academy, Lithuanian University of Health Sciences, Kaunas LT-50161, Lithuania; ^3^Department of Laboratory Medicine, Medical Academy, Lithuanian University of Health Sciences, Kaunas LT-50161, Lithuania

## Abstract

Atrial fibrillation (AF) despite the absence of heart failure is related to increased levels of natriuretic peptides (NPs). NPs have not been widely investigated in relation to left atrium (LA) function after sinus rhythm (SR) restoration and duration of AF. The aim of the study was to determine the changes of NPs levels and to define their relation with LA phasic function after electrical cardioversion (ECV).* Methods*. The study included 48 persistent AF patients with restored SR after ECV. NT-proANP and NT-proBNP were measured for all patients before the ECV. LA phasic function (*reservoir*,* conduit*, and* pump* phases) was assessed using echocardiographic volumetric analysis within the first 24 hours after ECV. Patients were repeatedly tested after 1 month in case of SR maintenance.* Results*. After 1 month, SR was maintained in 26 (54%) patients. For those patients, NT-proBNP decreased significantly (p=0.0001), whereas NT-proANP tended to decrease (p=0.13). Following 1 month after SR restoration, LA indexed volume decreased (p=0.0001) and all phases of LA function improved (p=<0.01). Patients with AF duration < 3 months had lower NT-proANP compared to patients with AF duration from 6 to 12 months (p = 0.005). Higher NT-proANP concentration before ECV was associated with lower LA* reservoir* function during the first day after SR restoration (R=-0.456, p=0.005), whereas higher NT-proBNP concentration after 1 month in SR was significantly related to lower LA* reservoir* function (R=-0.429, p=0.047).* Conclusions*. LA indexed volume, all phases of LA function, and NT-proBNP levels improved significantly following 1 month of SR restoration. Preliminary results suggest that higher baseline NT-proANP levels and higher NT-proBNP for patients with maintained SR for 1 month are related to lower LA* reservoir* function. The longer duration of persistent AF is associated with higher NT-proANP concentration.

## 1. Introduction

Atrial fibrillation (AF) is the most common arrhythmia affecting approximately 1% of the adult population in Europe [1]. AF is associated with 10–30% of all ischemic strokes and predicts worse neurological outcomes [2]. Left atrial (LA) size as a predictor of ischemic stroke was an object of studies for a long time but the exact relation has not been defined previously according to controversial results [3]. The concept of atrial cardiomyopathy as a progressive fibrotic atrial disease has been evolved in the recent years and this concept supports the perspective for AF to be a marker of increased stroke risk [4]. Investigations of LA functional reverse after sinus rhythm (SR) restoration become more important because LA dysfunction is known to be present despite normal LA size and SR [5].

LA also functions as a neurohumoral organ contributing to cardiovascular homeostasis by storing atrial natriuretic peptide (ANP) and small amounts of B-type natriuretic peptide (BNP) in the granules of atrial myocytes [6]. The natriuretic peptide (NP) system response is closely associated with myocardial tension and is known to be one of the criteria for the diagnosis of heart failure [7]. Concurrently, AF is related to increased levels of plasma NPs, even in the absence of congestive heart failure [8]. According to the 2016 ESC guidelines for heart failure management, the diagnosis of heart failure in patients with AF remains challenging and current diagnostic algorithm lacks specificity for those patients. There is a need for further investigation to establish cut-off values of NPs levels in patients with concomitant HF and AF [9]. Previous studies revealed that NPs concentrations are significantly reduced or normalized for patients with AF after the reversion to SR [10, 11]. However, the relation between LA phasic function and NPs levels after SR restoration has not been investigated previously.

The aim of this study was to determine the changes in N-terminal proatrial natriuretic peptide (NT-proANP) and N-terminal-proBNP (NT-proBNP) plasma levels and to define their relation with LA phasic function after the electrical cardioversion (ECV) of persistent AF.

## 2. Materials and Methods

### 2.1. Study Population

The prospective study included 66 patients with persistent AF from the screening group of 204 consecutive patients scheduled for ECV at Cardiology Department at Hospital of Lithuanian University of Health Sciences Kauno klinikos during the period of 2016–2017. 14 patients were excluded from the study before ECV because after initial tests exclusion criteria were confirmed (thyrotoxicosis (n=1); valvular AF (n=1); thrombus in LA (n=1), left ventricular ejection fraction (EF) <35% during AF (n=2)) or spontaneous conversion occurred until ECV (n=9). During the study period, we also excluded 4 participants whose ECV was unsuccessful (n=2) and whose diagnosis of heart failure was considered after ECV (n=2). Finally, the study group consisted of 48 patients with persistent nonvalvular AF. Inclusion criteria were as follows: adult patient >18 years of age of any gender, AF of any unknown duration, proven indication to restore SR by ECV, and signing an informed consent form. We excluded patients with relevant medical history of heart failure with preserved or reduced EF, newly diagnosed significant left ventricular dysfunction (left ventricular EF < 35%), confirmed ischemic heart disease, severe valvular heart disease, pacemaker, and other known diseases which could affect results of laboratory tests (severe renal failure and liver disease, use of glucocorticosteroids, any type of cancer, and thyroid disorders). At 1-month follow-up (FU) visit diagnosis of heart failure was reassessed for all patients with SR. In all cases, diagnosis of heart failure was made according to the 2016 ESC Guidelines for the diagnosis and treatment of acute and chronic heart failure [9]. The study protocol was approved by Kaunas Regional Biomedical Research Ethics Committee (ref. number: BE-10-4).

### 2.2. Follow-Up

All patients were followed up one month after the SR restoration. Individuals who developed such symptoms as palpitations or irregular heart activity were referred to an outpatient consultation of a cardiologist, when the ECG was performed and a detailed treatment plan was assigned for individual patient. Patient was referred to AF relapse group if the AF was confirmed. FU was performed in 1 month (28-35 days) after the SR restoration for asymptomatic patients. In case of registered AF in ECG, the investigated patient was assigned to the AF relapse group as well and no further tests were performed according to the study protocol. All patients, whom SR was registered on ECG at 1 month FU visit, were assigned to the SR group and two-dimensional (2D) echocardiography and NPs tests were done according to the protocol.

### 2.3. Echocardiographic Analysis

Baseline 2D echocardiography at rest was performed for all patients in 24 hours after ECV. The 2D echocardiography was repeated 1 month later in case of successful maintenance of SR. 2D transthoracic Doppler echocardiography was performed using the diagnostic ultrasound system (EPIQ 7, Phillips Ultrasound, Inc., Washington, USA). Standard 2D echocardiographic measurements were performed according to the Guidelines of the American Society of Echocardiography and analyzed by a single investigator [12]. LV EF was calculated using the modified Simpson's method. LA volumes were measured by the modified Simpson's disc summation method using apical four- and two-chamber views. LA maximum volume (Vmax) was measured at the end-systolic frame, just before the opening of the mitral valve. LA minimum volume (Vmin) was measured at the end-diastolic frame, at the mitral valve closure, while ‘‘pre-A” volume (Vpre A) was measured at the onset of the P wave on ECG. Vmax was indexed to the body surface area to derive the LA volume index (LAVI). Calculations of LA phasic functions were done using the following formulas [13]:

- LA* reservoir* function/LA total emptying fraction = (*Vmax* − *Vmin*)/*Vmax* × 100%;
- LA* conduit* function/LA passive emptying fraction = (*Vmax* − *VpreA*)/*Vmax* × 100%;
- LA* pump* function/LA active emptying fraction = (*VpreA* − *Vmin*)/*VpreA* × 100%.

### 2.4. Biochemical Analysis

Blood samples were drawn from a peripheral vein in heparinized tubes according to standardized blood collection procedure immediately before ECV and centrifuged and the plasmas were stored at –80°C until analysis.

The plasma NT-proANP concentrations were measured using Human NT-proANP ELISA Kit (Biomedica Immunoassay, Biomedica Medizinprodukte GmbH & Co KG, Austria). Assay detection limit was 635 ng/L, measurement ranges were 15–127000 ng/L, within-run precision was 5%, and between-run precision was 9%.

CE IVD labelled Human NT-ProBNP ELISA Kits (PathFast, LSI Medience Corporation, Japan) were used for the plasma NT-proBNP concentrations measurement. Assay detection limit was 15 ng/L, reference ranges were <15–128.3 ng/L, measurement ranges were 15–30000 ng/L, within-run precision was 4.7%, and between-run precision was 5.4%.

Following the Quality Specifications for B-Type Natriuretic Peptide Assays recommendations provided by Committee on Standardization of Markers of Cardiac Damage of the IFCC, the manufacturer's assay characteristics and results were recalculated from original units to ng/L (1 nmol/L=12700 ng/L for NT-proANP and 1 pg/mL=1 ng/L for NT-proBNP) [14].

Nowadays, researchers Apple FS and Clerico A with colleagues emphasize some limitations and difficulties in interpretation of NPs results because of the variety of immunoassay methods used for their measurement [14, 15]. Analyzing results is important to take into consideration the fact that assays for BNP and NT-proBNP (same for ANP and NT-proANP) may vary in their analytical sensitivity and specificity and susceptibility to analytical interferences due to cross-reactivity of structurally related NPs (ANP, BNP, CNP, pro and NT-pro components, and fragments of NPs) or other peptide hormones [15].

### 2.5. Statistical Analysis

Statistical analysis was performed using IBM® SPSS® Statistics 20 (Armonk, NY: IBM Corp., USA). Normal distribution of the continuous values was assessed by the Shapiro-Wilk test. The continuous variables are expressed as mean ± standard deviation or median (25th–75th confidence intervals). The categorical variables are expressed as absolute numbers and percentages.

Normally distributed continuous variables were compared using independent-samples and paired-samples* t*-tests. A Mann–Whitney rank-sum test and Wilcoxon test were used if data were not normally distributed. One-way ANOVA and Kruskal-Wallis test were used for comparing more than two independent samples. Scheffe test was used for post hoc multiple comparisons. Categorical variables were compared using the Chi-square test. Spearman's correlation coefficients were calculated for relations between LA phasic functions and different NPs. The size of analyzed difference (between baseline and follow-up) was considered to be significant if *β* ≤ 0.2 (i.e., the post hoc power of statistical test ≥ 0.8) as a type I error *α* = 0.05. A p value < 0.05 was considered significant.

## 3. Results

At 1-month FU, 26 patients (54%) were in SR and 22 (46%) in AF. The baseline characteristics of study participants according to AF status at 1 month after ECV are presented in Table 1. Patients with AF recurrence and maintained SR were similar in relation to age, sex, pre-ECV medical history, and initial medications use; however, more patients from SR group used amiodarone (p=0.003). SR and AF groups showed no significant differences considering initial echocardiographic findings, including LAVI (p=0.19), LA* reservoir* (p=0.85),* conduit* (p=0.67), and* pump* (p=0.54) functions and concentrations of NT-proANP (p=0.31) and NT-proBNP (p=0.96) (Table 1).

NT-proBNP decreased significantly in 1 month of successful SR maintenance (p=0.0001), while NT-proANP tended to improve during the FU (p=0.13). The median LAVI decreased significantly after 1 month in SR from 48.84 (41.45-57.09) to 41.52 (36.40-48.59) ml/m2 (p=0.0001), which in turn improved significantly LA* reservoir*,* conduit*, and* pump* functions (p<0.001). All changes of echocardiographic findings and NPs concentrations after 1 month in SR group are presented in Table 2.

Statistical analysis by one-way ANOVA model suggested that there was a significant interaction between duration of persistent AF before ECV and initial NT-proANP concentration (p = 0.005) but not related with NT-proBNP (p=0.67). Post hoc analysis showed statistically significant difference between NT-proANP levels for patients with AF <3 months compared to patients with AF from 6 to 12 months (p=0.006). Baseline NT-proANP and NT-proBNP concentrations according to the duration of persistent AF are described in Table 3.

**Figure 1:**
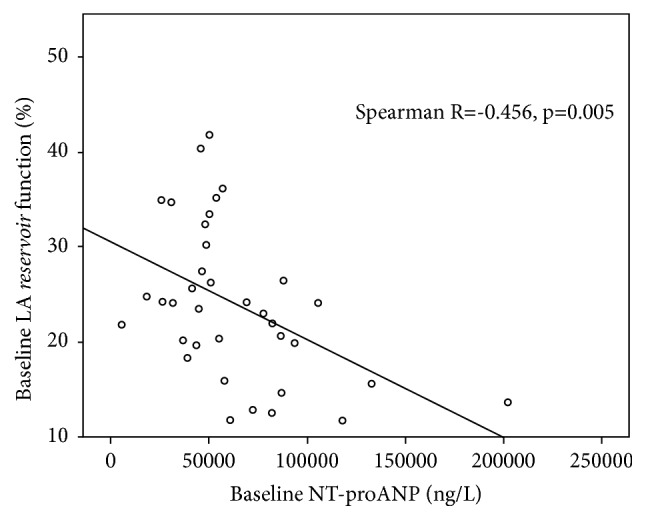
Correlation between NT-proANP concentration and left atrium* reservoir* function at baseline for the whole patient population.

**Figure 2:**
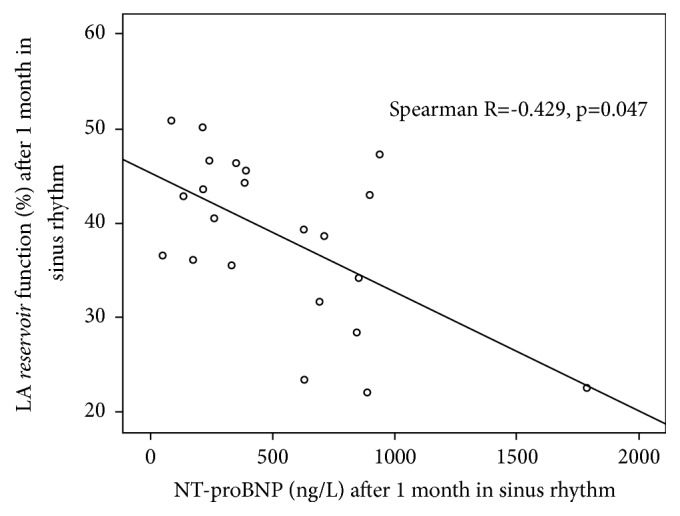
Correlation between NT-proBNP concentration and left atrium* reservoir* function for patients with SR maintenance.

Correlations between the LA* reservoir*,* conduit*, and* pump* functions and the NPs concentrations (NT-proANP and NT-proBNP) at baseline for the whole patient population and after 1 month for SR group were calculated. Baseline NT-proANP concentration had moderate negative correlation with the LA* reservoir* function during the first day after SR restoration (Spearman R = -0.456, p = 0.005), but no significant correlations were detected after 1 month in SR (Spearman R = 0.105, p = 0.69) (Figure 1). On the contrary, higher NT-proBNP concentration after 1 month in SR was significantly related to lower LA* reservoir* function (Spearman R = -0.429, p = 0.047) (Figure 2), and no significant correlations between baseline NT-proBNP and LA* reservoir* function on the first day after SR restoration were detected (Spearman R = -0.276, p = 0.09). No correlations between the LA* conduit* and* pump* functions and the plasma concentration of NPs were detected either on the first day or following 1 month after successful SR restoration by ECV.

## 4. Discussion

AF recurrence rates after ECV according to the data of published studies range from 63% to 84% in the first year [16, 17]. The incidence of AF recurrence after ECV in our study was 55.1%, which is consistent with results of published studies, taking into consideration shorter, just 1 month, FU duration in our cohort.

In this study, we observed reducing of LA volume, improving of LA function, and decreasing of NPs levels following 1 month of SR restoration by ECV. By our data, the LAVI but not LA anteroposterior diameter decreased significantly after 1 month in SR. Other authors reported LA dimensions to be decreasing within 1 month and decreased significantly in 3 months after restoration of SR [18, 19]. Baseline LA phasic function assessed by volumetric analysis was significantly altered immediately after ECV, although following 1 month of SR restoration we observed a significant improvement of all LA phases:* reservoir*,* conduit*, and* pump*. Some previous studies that used conventional echocardiographic parameters also showed that the maximum improvement in atrial function was observed at the end of the first month [20, 21]. Meanwhile Ozkan H et al. used strain analysis and demonstrated that atrial stunning can last longer than 1 month and it depends on AF duration [22]. Several studies have proven elevated plasma NPs concentrations in most patients with AF and significant drop following SR restoration by ECV, radiofrequency catheter ablation, or pulmonary vein isolation. It is known that 1 month is enough period of time for patients with persistent AF to observe significant decrease in NT-proBNP and NT-proANP levels [23–25]. Our study data showed statistically significant decrease of NT-proBNP and tendency of NT-proANP to be lower after 1 month in restored SR by ECV. We suggest that decrease of NT-proANP did not reach statistical significance because of small sample size.

We found that the longer duration of persistent AF is associated with higher NT-proANP concentration. Van den Berg MP with colleagues in the year 2004 made a statement that longstanding AF causes atrial structural remodeling, resulting in functional cell loss and reduced ANP production [26]. Since that time, the relation between the duration of AF and various forms of ANP concentrations was investigated particularly in patients with persistent AF and advanced heart failure [27]. In recent years, Legallois D with colleagues reported that midregional proANP plasma concentration significantly correlated with AF duration and also midregional proANP concentrations were significantly higher in patients presenting with AF of >48 hrs [28]. NT-proANP concentration for prediction of AF duration seems to be an interesting point for future studies.

The most intriguing finding of our study is that higher NPs levels can be associated with worse LA* reservoir* function at baseline and after 1 month in SR. According to data of our small study, different NPs do not have the same value to predict lower LA* reservoir* function at the different time frame following ECV. Higher baseline NT-proANP levels were associated with lower LA* reservoir* function soon after ECV, in contrast to higher NT-proBNP levels after 1 month following successful ECV. Complex assessment of NPs levels and LA phasic function has not been extensively analyzed. Only one study showed that NT-proBNP levels predict the deterioration of some phasic LA functions such as* reservoir *and* pump* in hypertrophic cardiomyopathy population [29]. There are no available data at present time explaining how recovery of LA function after SR restoration is related to changes of NPs levels. We tried to explain this finding pathophysiologically considering the mechanism of NPs production in patients with AF. ANP secretion is mainly regulated by mechanical stretching of the atria; therefore plasma ANP concentration is rapidly decreased following restoration of SR. This happens in conjunction with filling pressures because of improvement in atrial mechanical function. Most of ANP is stored in granules of atria myocytes and is released as an acute physiological response to increased atrial pressure [30]. Thus, correlation between higher NT-proANP concentration and lower LA* reservoir* function is more likely soon after ECV than after 1 month in SR. On the contrary, BNP has minimal storage in granules and most BNP is synthesized in bursts of activation from physiological and pathophysiological stimuli [30]. LA* reservoir* function is closely linked with LV mechanics. Ramkumar S with group of researchers showed that left ventricle global longitudinal strain was independently associated with LA* reservoi*r strain [31]. Relationship between LA* reservoir* function and NT-proBNP levels after 1 month in SR could be explained by this association. We highlight that we report a preliminary data from one study with small sample size and further researches (including our ongoing study) are needed to clarify the relationship between LA function and variation of different NPs following SR restoration.

## 5. Conclusions

Left atrium indexed volume, all phases of left atrium function, and NT-proBNP improved significantly following 1 month of sinus rhythm restoration. The longer duration of persistent AF is associated with higher NT-proANP concentration. According to preliminary results of this study, higher baseline NT-proANP levels and higher NT-proBNP for patients with maintained sinus rhythm for 1 month were related to lower left atrium* reservoir* function.

## 6. Limitations

The present study has several limitations. First, this was a single-center study with small number of patients. Second, we did not include LA strain analysis in our protocol, which could be more precise and accurate way for detecting the first asymptomatic signs of atrium dysfunction. Third, we chose to perform echocardiography within 24 hours after ECV in regular R-R intervals of SR, thus obviously limiting the clinical implications of our findings on the everyday clinical practice, when patients are imaged in AF. Larger studies are required to be performed to certify the possible relations between LA phasic function and NPs concentrations.

## Figures and Tables

**Table 1:** Patients' baseline characteristics according to rhythm status after 1 month.

	Total (n= 48)	SR group (n = 26)	AF group (n= 22)	p value
Age, years	65.15±10.19	64.04±9.66	66.45±10.88	0.424
Sex, males	33 (67.3%)	18 (69.2%)	14 (63.6%)	0.480
AF history				
<1 year	26 (53.1%)	15 (57.7%)	11 (50.0%)	0.433
1-3 years	8 (16.3%)	4 (15.4%)	4 (18.2%)	
>3 years	14 (28.6%)	7 (26.9%)	7 (31.8%)	
AF duration before ECV				
<3 months	27 (55.1%)	17 (65.4%)	10 (45.5%)	0.178
3-6 months	13 (26.4%)	5 (19.2%)	8 (36.4%)	0.405
6-12 months	8 (16.3%)	4 (15.4%)	4 (18.2%)	
Hypertension	42 (85.7%)	22 (84.6%)	20 (90.9%)	0.758
Diabetes	5 (10.2%)	5 (19.2%)	0	

*Echocardiographic parameters within the first 24 hours after SR restoration*				
Left ventricular ejection fraction, %	53.76±11.82	52.84±13.46	54.78±9.99	0.67
Left ventricular end diastolic diameter, mm	50.72±4.15	51.29±3.49	50.07±4.81	0.28
LA diameter, mm	48.66±4.26	48.21±4.09	49.17±4.52	0.61
LA volume, ml/m^2^	51.85 (45.34-57.78)	49.94 (39.89-57.09)	52.10 (47.94-61.71)	0.19
LA *reservoir* function, %	24.16 (19.99-33.21)	24.79 (18.56-35.16)	24.12 (24.12-32.95)	0.85
LA *conduit* function, %	15.88±6.33	16.51±7.48	15.94±5.68	0.67
LA *pump* function, %	9.67 (6.97-14.88)	8.47 (6.27-15.02)	10.34 (7.63-13.74)	0.54

*Medications*				
Propafenone	10 (20.4%)	2 (7.4%)	8 (36.4%)	0.06
Amiodarone	19 (38.8%)	16 (59.3%)	3 (13.6%)	0.003
*β*-blocker	45 (91.8%)	24 (88.9%)	21 (95.5%)	0.66
ACEI or ARB	37 (75.5%)	20 (74.1%)	17 (77.3%)	0.62
Statins	10 (20.4%)	6 (22.2%)	4 (18.2%)	0.53

*Plasma levels of natriuretic peptides*				
NT-proBNP, ng/L	1763.50 (1030.25-2556.00)	2276.00 (918.00-3186.00)	1670.00 (1119.00-2280.25)	0.96
NT-proANP, ng/L	51181.00 (39243.00-82423.00)	55626.00 (41052.75-83724.75)	48133.00 (33972.50-73533.00)	0.31

**Table 2:** Natriuretic peptides concentrations and echocardiographic parameters after 1 month in sinus rhythm group.

	Baseline	Follow-up	p value	*β*
NT-proBNP, ng/L	2276.00 (918.00-3186.00)	631.00 (242.00-853.00)	**0.0001** **∗**	**0.0** **∗** **∗**
NT-proANP, ng/L	55626.00 (41052.75-83724.75)	40640.00 (33147.00-71501.00)	0.13	-
LVEF, %	53.04±13.16	54.01±8.36	0.62	-
LVEDD, mm	51.26±3.59	50.32±3.49	0.23	-
LA diameter, mm	47.66±3.43	44.60±8.55	0.15	-
LA volume, ml/m^2^	48.84 (41.45-57.09)	41.52 (36.40-48.59)	**0.0001** **∗**	**0.001** **∗** **∗**
LA *reservoir* function, %	25.22 (17.63-35.42)	41.68 (35.22-46.42)	**0.0001** **∗**	**0.0** **∗** **∗**
LA *conduit* function, %	16.09 (11.99-19.45)	19.29 (15.86-23.21)	**0.006** **∗**	**0.154** **∗** **∗**
LA *pump* function, %	9.26 (6.67-16.68)	23.43 (17.64-31.79)	**0.001** **∗**	**0.004** **∗** **∗**

*∗* statistically significant difference; *∗∗* computed as *α* = 0.05.

**Table 3:** Natriuretic peptides concentrations according to atrial fibrillation duration prior to electrical cardioversion.

	*AF episode prior to ECV*			p value	*β*
	<3 months	3-6 months	6-12 months		
	(n=27 pts)	(n=13 pts)	(n=8 pts)		
NT-proANP, ng/L	46724.96±24536.20#	66399.83±31630.25	92202.00±49715.92	**0.005** **∗**	**0.133** **∗** **∗**
NT-proBNP, ng/L	1672.00 (998.25-2283.00)	1573.00 (1035.50-3936.50)	1710.00 (584.00-3508.00)	0.67	-

*∗* statistically significant difference; *∗∗* computed as *α* = 0.05; # p = 0.001 compared to 6-12 months.

## Data Availability

The data supporting the conclusions of this article are available from the corresponding author upon reasonable request due to privacy restrictions.

## Abbreviations

AF: Atrial fibrillation
LA: Left atrial
SR: Sinus rhythm
ANP: Atrial natriuretic peptide
BNP: B-type natriuretic peptide
NP: Natriuretic peptide
NT-proANP: N-terminal proatrial natriuretic peptide
NT-proBNP: N-terminal-pro-BNP
ECV: Electrical cardioversion
LV EF: Left ventricular ejection fraction
FU: Follow-up
2D: Two-dimensional
Vmax: LA maximum volume
Vmin: LA minimum volume
Vpre A: ‘‘pre-A” volume
LAVI: Left atrium volume index
ACEI: Angiotensin-converting enzyme inhibitors
ARB: Angiotensin II receptor blockers
LVEDD: Left ventricular end-diastolic diameter.
